# Risk Recognition and Multidisciplinary Approach for Non-Cardiac Surgeries in Paediatric Cardiac Patients: A Retrospective Observational Study

**DOI:** 10.7759/cureus.12030

**Published:** 2020-12-11

**Authors:** Poonam Motiani, Vibha Chhabra, Zainab Ahmad, Pramod K Sharma, Anju Gupta

**Affiliations:** 1 Paediatric Anaesthesia, Super Speciality Paediatric Hospital & Post Graduate Teaching Institute, Noida, IND; 2 Anesthesiology and Critical Care, All India Institute of Medical Sciences, Bhopal, IND; 3 Paediatric Surgery, All India Institute of Medical Sciences, Bhopal, IND; 4 Anesthesiology and Critical Care, All India Institute of Medical Sciences, New Delhi, IND

**Keywords:** congenital heart disease, non cardiac surgery, paediatric, cardiac risk, anaesthesia

## Abstract

Background

Congenital heart disease (CHD), a structural and functional heart disease, is the commonest birth defect with an incidence of one in 125 live births worldwide with ventricular septal defect (VSD), atrial septal defect (ASD) and tetralogy of Fallot (TOF) constituting the majority. Surgery for associated extra-cardiac anomalies (airway, skeletal, genitourinary, and gastrointestinal) may be required in 30% of these patients. Delivery of uneventful anaesthesia in these children requires an understanding of not only paediatric anaesthesia but also of the pathophysiology of the cardiac lesion and its associated risks.

Aims

The purpose of this retrospective review was to highlight the approach to the anaesthetic management and outcomes of patients with significant cardiac lesions presenting for non-cardiac surgeries.

Material and methods

A retrospective chart review of all children with congenital heart disease (CHD) (repaired or unrepaired) who were posted for a non-cardiac surgery in this tertiary care Paediatric super-specialty hospital from January 1, 2018 to December 31, 2019 was carried out. Data on demographics, peri-operative management, and clinical course was retrieved.

Inclusion criteria were paediatric patients (0-18 years) of either gender with a diagnosis of a CHD (repaired or unrepaired) undergoing any non-cardiac surgeries (NCS) under anaesthesia/Monitored Anaesthesia Care (MAC).

Exclusion criteria were procedures only under local anaesthesia (LA) or a minor procedure done solely under sedation not involving an anaesthesiologist.

Results

During the study period, we found five eligible cases who underwent a total of six procedures. Five procedures were elective and one was an emergency. Preoperative optimization was conducted by a multidisciplinary team including paediatric surgeons, anaesthesiologists, physicians, and cardio-thoracic surgeons. Anaesthesia was conducted by at least a consultant paediatric anaesthesiologist. Overall all patients tolerated anaesthesia well without any adverse events or complications. All six anaesthetic encounters were safe and uneventful.

## Introduction

Congenital heart disease (CHD) is a common birth defect, with a global incidence of one in 125 live births [1]. The incidence has generally been higher in developing countries as compared to most developed countries e.g., the incidence in Central African Republic was found to be 3.38/100 in a study on the epidemiology of CHD, whereas the incidence in France which is a developed nation was found to be 0.86/100 [1]. In India, the prevalence of CHD was reported to be around 1.91/100 live births, and the commonest lesions were ventricular septal defect (VSD) at 33%, followed by atrial septal defect (19%) and tetralogy of Fallot (16%) [2]. Thirty percent of these children might require surgery during infancy due to associated extracardiac anomalies [3] and 85% of these are expected to survive to adulthood in the USA [4]. The challenge for anaesthesiologists in handling patients with CHD coming for non-cardiac surgeries (NCS) is due to the patient’s age, complexity of the heart lesion, risk recognition, defining goals of anaesthetic management, and multiple coexisting diseases. Paediatric patients with CHD undergoing NCS are associated with a two-fold increase in mortality [5]. The data from the Pediatric Perioperative Cardiac Arrest (POCA) registry, collected from 1994-2005, on 373 anaesthesia-related cardiac arrests, showed 34% to have a congenital or acquired heart disease. Also, 54% of the cardiac arrests (CA) in patients with heart disease (HD) occurred in the general operating room compared with 26% in the cardiac operating room. The authors concluded that the identification of causes of and factors relating to anesthesia-related CA suggests possible strategies for prevention [6]. However, Walker et al. reported that despite these challenges, neonates with complex cardiac disease requiring major surgery tolerate general anaesthesia (GA) with few complications [7].

Literature on the anaesthetic management in this regard is still deficient in some aspects and for various anomalies and is largely based on studies of individual cases, so there is a need to categorise the risk status and define peri-operative concerns and goals of management for CHD patients undergoing NCS [8]. In this retrospective observational study, we aimed to review the demographics, peri-operative management and clinical course of paediatric patients with CHD (uncorrected/corrected) presenting for NCS, retrospectively, to highlight the approach to the peri-operative anaesthetic management of these patients undergoing NCS in a developing country.

## Materials and methods

This single-center retrospective study was conducted at Super Speciality Paediatric Hospital and Postgraduate Teaching Institute, Noida, Uttar Pradesh, India after Institutional Ethics Committee approval (2019-10-IM-01). It included all children with CHD (repaired, palliated, or unrepaired) who were posted for an NCS from January 1, 2018 to December 31, 2019. Data was collected using local electronic operation records, operation theatre (OT) registers and archived case records. OT registers were searched manually, and electronic operation records were searched using the Windows search function for the terms ‘cardiac’/‘non-cardiac’. Three investigators (PM, PKS, and VC) carried out the data collection from local and electronic operation records and resolved the queries by searching the surgical case records, investigations, and questioning the pediatricians, surgeons, and parents. The other two investigators (ZA and AG) carried out the data analysis and literature search. Our inclusion criteria were paediatric patients (0-18 years) of either gender with a diagnosis of a CHD (repaired or unrepaired) undergoing any NCS, under general anaesthesia (GA)/monitored anaesthesia care (MAC). Exclusion criteria were surgical or diagnostic procedures done only under local anaesthesia (LA), or a minor procedure done solely under sedation, not involving an anaesthesiologist.

Data regarding demography and patient characteristics like age, gender, weight, type and severity of cardiac lesion, diagnosis, and surgery were retrieved. The records were reviewed for all clinical details including preoperative anaesthetic evaluation, investigations, optimization, intraoperative management, and post-operative follow-up till hospital discharge. Data retrieved included type of anaesthesia, operative and anaesthetic time, airway management, hemodynamic stability, need for vasoactive and anti-arrhythmic drugs, and any intra-operative or post-operative complications. The children were also categorized into High, Intermediate, and Low risk, using factors such as physiologic status, disease complexity, type of surgery, and age, as given by White et al. [9]. Physiologically poorly compensated patients with presence of major complications like cardiac failure, pulmonary hypertension, arrhythmias or cyanosis, presence of complex lesions (single-ventricle or balanced circulation physiology, cardiomyopathy, aortic stenosis), major surgery (intraperitoneal, intrathoracic, with anticipated major blood loss requiring transfusion), < two yrs of age, emergency surgery, preoperative hospital stay more than 10 days and American Society of Anesthesiologists (ASA) physical status IV or V were as defined High-risk patients [9].

The data thus collected was entered into a standardized data collection form (Microsoft Excel) and all queries were resolved by discussion with senior members of the team. Only descriptive statistics was used. The detailed clinical management of these patients has been discussed.

## Results

In this retrospective chart review, eight surgical cases were found eligible, out of which two were excluded as they were conducted under local anaesthesia by the surgeons (one was a dental extraction in a 15-year-old boy and another was a minor wound debridement in a 14-year-old girl).

We found that during the study period, six surgical procedures conducted on five CHD patients were eligible for inclusion in the study and were enrolled. Out of these, five procedures were elective and one was an emergency. Five procedures were on female patients, and one was done in male child. Mean (SD) for age and weight were 3.8 (9.9) years and 12.68 (4.6) kg. The CHD was a right to left shunt (cyanotic) in four cases and acyanotic in two cases [left to right in one case and no shunt (aortic stenosis) in one case]. On risk stratification, three cases were at high cardiac risk status, two at intermediate risk, and one at low risk status. One was a repaired CHD (post atrial septal defect repair), four were palliated (two cases with Glenn shunt, one Post Blalock-Taussig (BT) shunt and one post aortic valve balloon dilatation) and one was unrepaired tetralogy of Fallot (TOF). Patient characteristics and peri-operative data is depicted in Table 1. Anaesthesia was conducted by at least one consultant paediatric anaesthesiologist. Ketamine was used as induction agent in all cases and sevoflurane in O_2_/Air mixture was used for maintenance. Multimodal analgesia was prescribed in majority of the cases. Overall, all patients tolerated anaesthesia well without any adverse events or complications. These cases are described individually in greater detail below.

**Table 1:** Patient characteristics and perioperative data of children with congenital heart disease (CHD) undergoing non-cardiac surgery (NCS) AA=anaesthetic agent, ACE=angiotensin converting enzyme, AS=aortic stenosis, ASA PS=American Society of Anaesthesiologists physical status, AV=aortic valve, BIS=bi-spectral index, BT=Blalock Taussig, CETT=cuffed endotracheal tube, CxR=chest X-ray, FM=face mask, GA=general anaesthesia, Hb=haemoglobin, IABP=invasive blood pressure, IE=infective endocarditis, IPPV=intermittent positive pressure ventilation, L-R=left to right, LV=left ventricle, LVH=left ventricular hypertrophy, MAC=monitored anaesthesia care, MOS=morning of surgery, NIBP=non-invasive blood pressure, NPO=nil per orally, NSAIDs=nonsteroidal anti-inflammatory drugs, ORA=on room air, PBF=pulmonary blood flow, PDA=patent ductus arteriosus, PEEP=positive end expiratory pressure, PVR=pulmonary vascular resistance, R-L=right to left, RVOT=right ventricular outflow tract, SpO2=oxygen saturation, SVR=systemic vascular resistance, TAPVC=total anomalous pulmonary venous connection, TEF=tracheoesophageal fistula, TOF=tetrology of Fallot, TV=tidal volume, URI=upper respiratory infection, VCV=volume-controlled ventilation, VSD=ventricular septal defect

Case No.	Age, Gender, Weight; Presenting Complaint	Significant on Preoperative Evaluation	Sequence of events	Risk stratification for NCS; ASA PS Classification; Preoperative Advice	Specific perioperative Concerns and preparation for NCS	Anaesthesia Technique
1	2yr, F, 9 kg; Nasal defect	-Frequent URI -Poor weight gain - dyspnea; -CxR: Cardiomegaly, pulmonary plethora. -Echo: PDA 3-4mm, L-R shunt, normal LV function	-PDA ligation surgery done - Nasal defect correction surgery a month later	-Low risk [9]; -ASA PS II; -Continue ACE inhibitors and diuretics till MOS.-Oral Midazolam	- General Perioperative concerns (Table 3); -Routine	General Anaesthesia (GA): -IV Induction: Ketamine and fentanyl; NMB: Vecuronium, -Maintenance: Sevoflurane in O_2_/Air - Analgesia: PCM, NSAIDs
2	11 yr, F, 28 kg; Microtia (Figure 2)	-Follow up of CHD with TAPVC, operated six years ago; Glenn’s stent (in situ) -SpO_2_ :89% ORA -Hb: 18.9 g/dL -Echo: Patent Stent	- Ear reconstruction surgery, with Dectopectoral flap	-Intermediate risk [9]; - ASA PS II; - Continue Antiplatelets, -Oral Midazolam	-Cyanosis, poor cardiorespiratory reserve, ongoing hypoxemia, polycythemia, hyperviscosity of blood and altered coagulation profile.	GA: IABP monitoring -IV Induction: Ketamine and fentanyl; NMB: Vecuronium, -Maintenance: Isoflurane in O_2_/Air; -Analgesia :PEC-I block ,PCM,NSAIDS
3	9 yr, M, 19 kg; -Bleeding per rectum x 3 d -weakness	-Listless, Pallor (Hb-5.75 g/dL), -Tachycardia -Hypotension -History of syncopal attacks, poor weight gain, easy fatigability -Severe valvular AS, with bicuspid aortic valve and LVH detected.	-AV balloon dilatation -Exploratory Laparotomy one month later (emergency)	- High Risk [9]; -ASA PS IV_E _; -Antibiotic prophylaxis -IV Midazolam premedication	-Avoid Myocardial ischaemia, maintain SVR , Invasive monitoring; -Blood and fluid resuscitation, minimal sedative premedication, and minimal fasting -Psychological support and Informed high-risk consent.	GA IABP and CVP monitoring -IV Induction: Ketamine and fentanyl; NMB: Vecuronium, -Maintenance: Sevoflurane in O_2_/Air; -Analgesia: wound infiltration, PCM, NSAIDs
4	45-day, F, 2.5 kg; Post TEF Repair with TOF.	-2D Echo:VSD, R-L Shunt,Severe RVOT obstruction, small PDA - SpO_2 _ORA 80-84% -Hb-19 g/dL.	- Oesophageal dilatation; - Followed by TOF repair 5 days later	- High Risk [9]; -ASA PS IV ; -Continue IV fluids -No sedative premedication	-Balance PVR and SVR, Prevent increase in R-L shunt, prevent hypercyanotic spells; polycythemia and coagulopathy; -Prefer I.V induction - ABG monitoring essential. -Adequate pain relief	GA -Preoxygenation, anticholinergics -Induction fentanyl and ketamine. -NMB (Atracurium) - Maintenance: Sevoflurane in O_2_/air -Analgesia: PCM
5	3 months, F 2.9 kg; Post BTshunt, Persistent feeding difficulty	- SpO_2_ ORA 84-86%, -Hb-13.7 g/dL	-Redo-oesophageal dilatation	-High Risk [9]; -ASA PS IV; -Continue IV fluids -No sedative premedication	- Concerns as in Case No.4 In addition: -Greater risk to child, cyanosis, hypoxia, poor weight gain, thrombosed veins, and fourth GA exposure.	GA Induction: Fentanyl and Ketamine; NMB; -Maintenance: Sevoflurane in O_2_/Air -Analgesia: PCM
6	6 yr, F, 15 kg; Definitive cardiac surgery for Cyanotic Heart disease	-Cyanotic CHD, with Glenn’s shunt (in situ) - SpO_2_ 85% ORA - Hb 17 g/dL - Echo: Patent stent. - Patient on antiplatelet therapy	-Tooth extraction -Definitive cardiac surgery 5 days later.	- Intermediate risk [9]; -ASA II -IE prophylaxis -Antiplatelets continued -Oral sedative premedication	-Risk of I.E, Cyanosis, nil cardiorespiratory reserve, ongoing hypoxemia, polycythemia, risk of hyperviscosity and thrombosis; Avoidance of rise in PBF and fall in SVR.	GA: - Inj Ketamine and Inj Midazolam; -Anticholinergic -Spontaneous ventilation on FM - Intraoperative: vitals stable - SPO_2_ 92-94% Analgesia; Inj PCM, wound infiltration

Case 1

**Figure 1:**
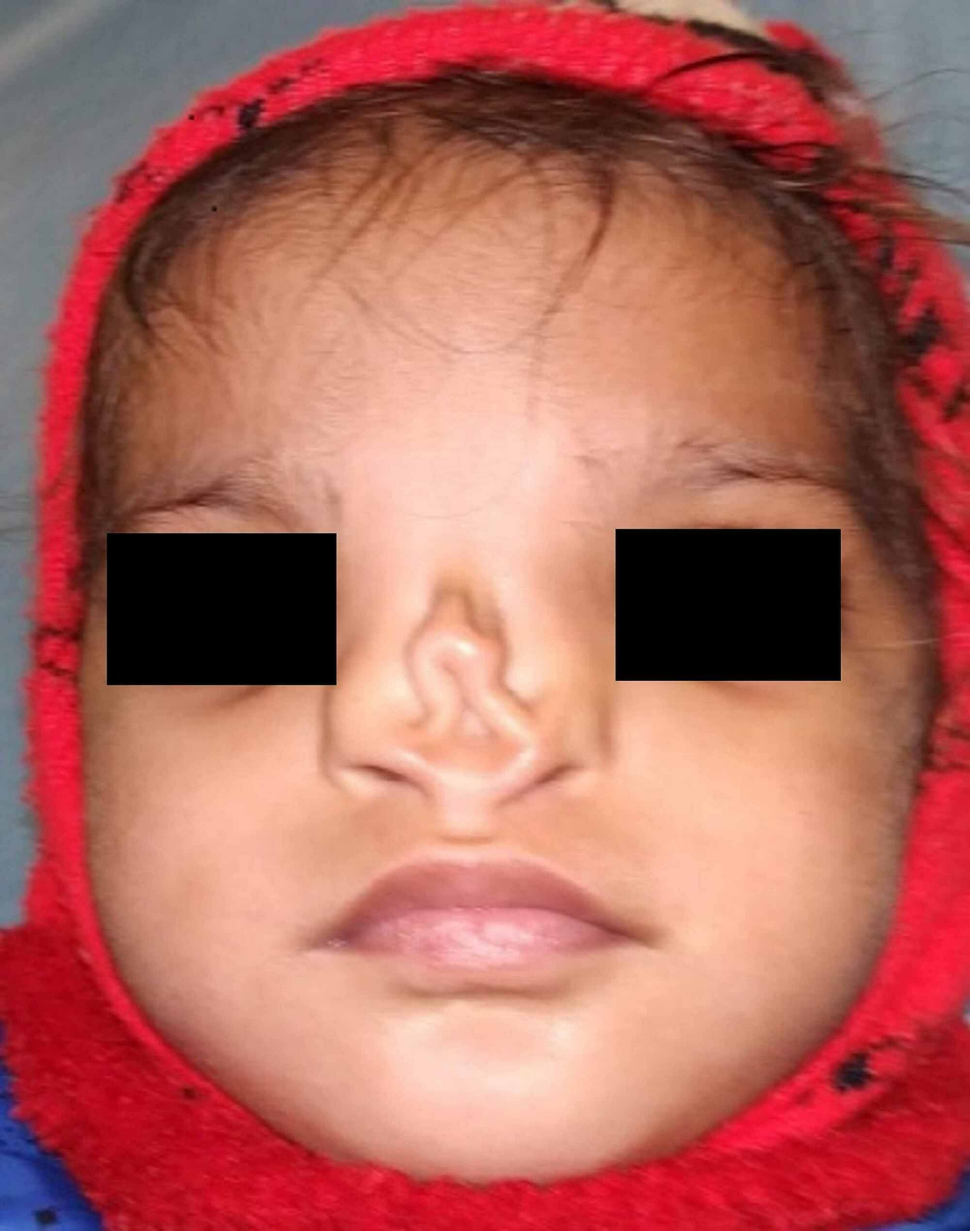
Two-year-old female, with undetected PDA, planned for nasal defect correction surgery. PDA: Patent Ductus Arteriosus

A two-year-old girl weighing 9 kg was planned for congenital nasal defect correction surgery (Figure 1). On routine pre-anaesthesia evaluation, significant history of frequent upper respiratory infection (URI), poor weight gain and dyspnea prompted us to investigate the child further. Chest X-ray (CXR) was done, and it revealed cardiomegaly and pulmonary plethora. Further, on echocardiography, a patent ductus arteriosus (PDA) 3-4mm, with left to right shunt, gradient 66 mmHg with normal left ventricular (LV) function was detected. After cardiology and cardiac surgery consultations, the patient was started on angiotensin-converting enzyme (ACE) inhibitors and diuretics, and first underwent PDA ligation surgery uneventfully and was dated for nasal defect correction surgery a month later. 

A month later, in the pre-anaesthesia checkup (PAC) clinic, her systemic examination and laboratory investigations including 2D-Echo were unremarkable. The patient was assigned ‘Low risk’ according to the risk stratification of children with CHD undergoing NCS [9] and accepted under ASA PS II. She was advised to continue ACE inhibitors and diuretics till morning of surgery.

After IV midazolam premedication, the patient was shifted to the operating room (OR). Routine ASA monitoring was instituted. Following anaesthesia induction with titrated doses of ketamine and fentanyl and neuromuscular blockade (NMB) with vecuronium, the patient’s trachea was intubated with a cuffed endotracheal tube (CETT) #4.0. Anaesthesia was maintained with sevoflurane in oxygen (O_2_)/air mixture, intermitted doses of vecuronium and controlled ventilation. Further course including reversal of neuromuscular blockade, emergence and post-operative recovery was uneventful.

Case 2

**Figure 2:**
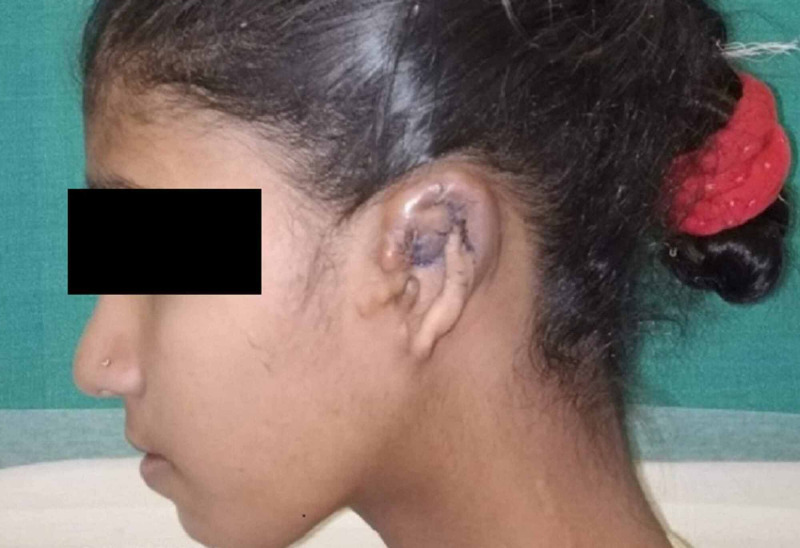
11-year-old female, CHD with TAPVC with a Glenn’s Shunt in situ for ear reconstruction surgery. CHD: Congenital Heart Disease, TAPVC: Total Anomalous Pulmonary Venous Connection

An 11-year-old girl, weighing 28 kg, a follow-up case of CHD with total anomalous pulmonary venous connection (TAPVC), operated six years ago with a Glenn’s stent (in situ) was planned for ear reconstruction surgery with decto-pectoral flap (Figure 2). History, examination and investigations on pre-anaesthetic evaluation revealed baseline oxygen saturation (SpO_2_) of 89% on room air (ORA) and hemoglobin (Hb) of 18.6 g/dL. Other investigations including electrocardiogram (ECG) and CXR were normal. Echocardiography showed a patent stent. She was continued on anti-platelet medication, accepted for anaesthesia under ASA II, and placed in ‘Intermediate risk’ category [9]. Clear fluids were allowed till two hours pre-operatively. Oral midazolam pre-medication was given.

In the OR, standard non-invasive monitoring along with invasive arterial blood pressure (IABP) monitoring was instituted. GA was induced with incremental doses of ketamine and fentanyl and trachea was intubated with a CETT # 5.5 following NMB with vecuronium. Maintenance of anaesthesia was with isoflurane in O_2_/air; injection fentanyl and vecuronium top-ups were administered hourly. Intra-operatively, the vitals remained stable and SpO_2_ ranged from 93-96%. At the end of surgery lasting for three hours, an ultrasound-guided pectoralis nerve block (PEC-I) was given for postoperative pain relief. Reversal of neuromuscular blockade and emergence were uneventful. Following an uneventful post-operative course, the patient was discharged at post-operative day five.

Case 3

A six-year-old boy weighing 19 kg presented to the hospital emergency with history of bleeding from rectum for three days and generalized weakness. The patient was listless, very pale (Hb 5.75 g/dL), tachycardic (HR 130/min), and hypotensive (BP 78/46 mmHg).

On pre-anaesthesia evaluation, parents gave history of syncopal attacks, poor weight gain and easy fatigability. A mid-systolic murmur was auscultated in the aortic area. On further evaluation, severe valvular aortic stenosis (AS) with bicuspid aortic valve and concentric left ventricular hypertrophy (LVH) was detected. The patient underwent an uneventful aortic valve (AV) balloon dilatation for the same. 

A month later, the patient again presented with an episode of malaena and was posted for excision of Meckel’s diverticulum. On examination, the patient was conscious, but pale and tachycardic. Blood and fluid resuscitation were initiated. On investigation, anaemia (Hb 6.8 g/dL) was present. The patient was accepted under ASA IV-E and ‘High risk’.

Pre-operatively, the child received blood and fluid resuscitation, antibiotic prophylaxis, small dose of midazolam and was allowed clear liquids till two hours preoperatively.

In the OR, standard non-invasive monitoring along with central venous pressure (CVP) and IABP monitoring was instituted. GA was induced with incremental doses of IV ketamine along with fentanyl and vecuronium. The patient’s trachea was intubated with CETT #5.0mm. Anaesthesia maintenance was with sevoflurane, in O_2_/air 50/50 with intermittent boluses of NMB. Blood and blood products were administered intra-operatively, as per need and protocol. Volume controlled ventilation using low tidal volume and positive end-expiratory pressure (PEEP) was used to maintain a SpO_2_ 99-100% and an end-tidal CO_2_ concentration (EtCO_2_) of 40 mmHg. The surgery lasted three hours, was uneventful and blood gases were acceptable. Analgesia was provided with wound infiltration and routine scheduled doses of PCM and nonsteroidal anti-inflammatory drugs (NSAIDs). The patient’s trachea was extubated in the OR and the child was discharged from the hospital after five days.

Case 4

A 2.5 kg, 45-day-old female child, post-tracheo oesophageal fistula (TEF) repair (repair done at day two of life), with tetralogy of fallot (TOF), presented to the hospital for TOF repair. 2D Echo revealed a VSD with aortic override (R-L Shunt), severe right ventricular outflow tract (RVOT) obstruction, adequate pulmonary artery (PA) flow, small PDA and normal coronaries. The SpO_2_ ORA was 80-84% and Hb 19 g/dL. Other investigations including coagulation parameters were normal. Patient also had an anastomotic leak following the TEF surgery. A nasogastric tube couldn’t be passed which suggested persistent oesophageal narrowing, for which esophageal dilatation was planned, prior to the cardiac surgery. The patient was accepted for anaesthesia under ASA-IV and in ‘High risk’ category [9]. Preoperatively IV fluids were continued, no sedative premedication was given.

**Figure 3:**
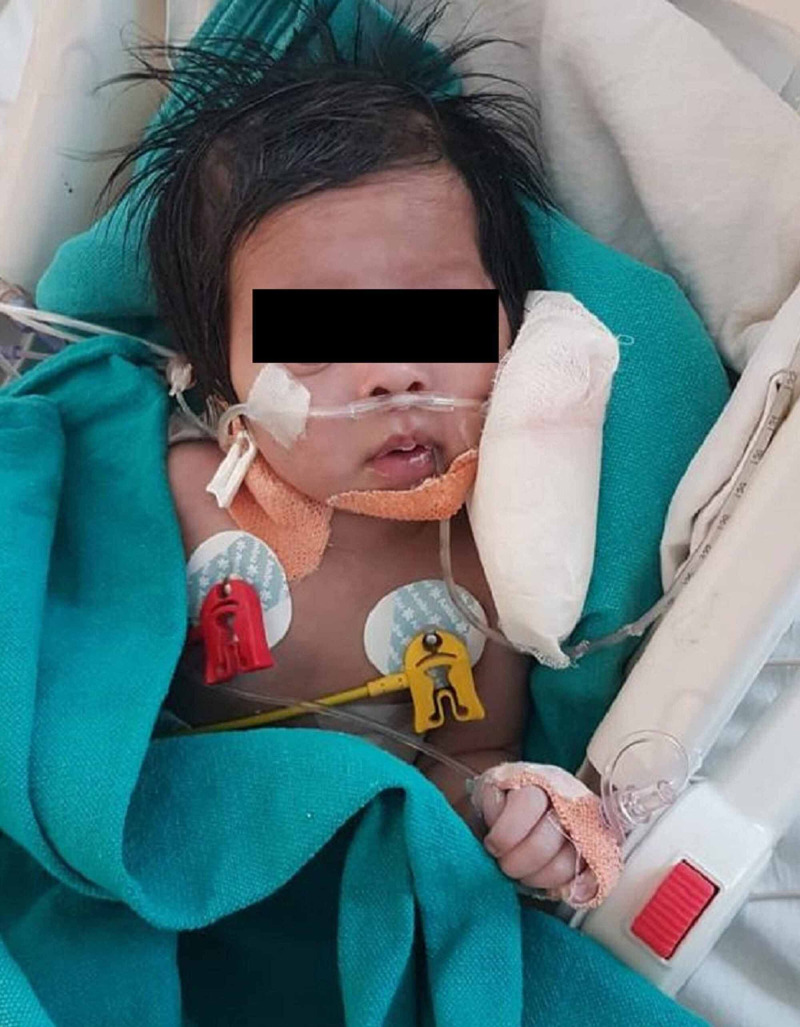
45-day-old female child, postoperative c/o tracheo oesophageal fistula (TEF) repair, with tetralogy of Fallot (TOF), after oesophageal dilatation

In the OR, ECG, SpO_2_ and non-invasive blood pressure (NIBP), EtCO2, temperature, bispectral index (BIS), and anaesthetic agent (AA) monitoring were instituted. Following pre-oxygenation and anticholinergic premedication, anaesthesia was induced with fentanyl and ketamine. After ensuring adequate mask ventilation, neuromuscular blockade (NMB) was achieved with injections of atracurium and videolaryngoscope-assisted trachea intubation done. Anaesthesia maintenance was with sevoflurane in O_2_/air mixture. Intra-operatively, the vitals remained stable; SpO_2_ varied from 80-88%, and following an uneventful further course, the neonate was extubated on table (Figure 3). The child received PCM for analgesia postoperatively.

Case 5

The same child described under Case 4 above, now 2.6 kg at three months, post Blalock-Taussig (BT) shunt for TOF (operated at day 50 of life), was readmitted due to persistent feeding difficulties, a month after the BT shunt surgery and planned for redo-oesophageal dilatation.

The SpO_2_ ORA was 84-86%, Hb 13.7 g/dL and other investigations were normal. The child was accepted for anaesthesia under ASA-IV, placed in ‘High risk’ category [9]. Pre-operatively, IV fluids were continued, no sedative premedication was given.

In the OR, availability of routine, emergency drugs and equipment and the presence of two paediatric anaesthesiologists, was ensured. Standard non-invasive monitoring was instituted. Child was premedicated with anticholinergics, anaesthesia was induced with injected fentanyl and injected ketamine in incremental doses, and the patient’s trachea was intubated after muscle relaxation with vecuronium. Sevoflurane in O_2_/air was used for maintenance of anaesthesia. Intra-operatively, vitals remained stable, SpO_2_ varied from 80-88% and rest of parameters was normal. PCM was given for analgesia and after an uneventful course, the neonate was extubated on table.

Case 6

A 15 kg, six-year-old girl with a cyanotic CHD, with a Glenn’s shunt (in situ) placed for a hypoplastic ventricle, was planned for a tooth extraction prior to a definitive cardiac surgery. Pre-anaesthetic evaluation revealed ongoing hypoxemia (SpO_2_ 85% ORA), polycythemia (Hb 17g/dL) and patient on anti-platelet therapy. Other investigations were essentially normal. Echocardiography showed a patent stent. Infective endocarditis prophylaxis was started, anti-platelets continued and patient accepted under ASA II and with ‘Intermediate risk’ [9]. Clear fluids were continued till two hours preoperatively and oral sedative premedication were advised.

After institution of standard ASA monitoring in the OR, and an anticholinergic, anaesthesia was induced with IV ketamine and midazolam and maintained on spontaneous ventilation with sevoflurane/O_2_ with face mask. Intra-operatively, the vitals remained stable, SpO_2_ ranged from 92-94% and the patient received IV and local anaesthetic infiltration for analgesia. After a further uneventful intraoperative and postoperative course, the patient was planned for a definitive cardiac surgery after five days.

## Discussion

Children with CHD have a significant incremental risk when presenting for minor or major surgery. The key considerations during preoperative optimization and management of these children have been summed up in Table 2. We report the peri-operative medical and anaesthetic management of six paediatric cardiac patients undergoing NCS, who were having different risk categorizations. Children with CHD presenting for noncardiac surgery have been grouped into: 1. Nonoperated patient; 2. Previous palliative surgery; 3. Previous corrective surgery [10].

**Table 2:** Key points in preoperative anaesthesia evaluation and perioperative management ACE=angiotensin converting enzyme, GA=general anaesthesia, NCS=non-cardiac surgery, PVR=pulmonary vascular resistance, SVR=systemic vascular resistance, URI=upper respiratory infection

Key considerations during preoperative optimization and management of these children	
1	Anaesthesiologists involved in management of these patients should have a proficient knowledge of the pathophysiology of the cardiac lesions and the significance of changes in SVR/PVR. Risk associated with NCS should be categorized depending on physiological status, disease complexity, type of surgery and age.
2	A thorough preoperative evaluation, focusing on history, physical examination and investigations, details of previous cardiac or NCS, need for prolonged intubation, medication history which may include aspirin, warfarin, antidepressants, diuretics, ACE inhibitors, and antiarrhythmics.
3	Associated congenital anomalies
4	Multidisciplinary approach usually required.
5	Recent or current URI, poorly tolerated by children with reduced pulmonary compliance, particularly those with a Glenn Shunt.
6	Venous access may be problematic, particularly, in children who have had peripheral or central lines in the past. Dehydration to be avoided.
7	Most cardiac medications should be continued preoperatively, including aspirin to prevent shunt thrombosis; Infective Endocarditis prophylaxis, as indicated.
8	Perioperative management of hypoxemia, cyanosis, polycythemia, shunt, pulmonary hypertension and ventricular dysfunction, if present.
9	Psychological preparation of the patient and the family.
10	Sympathetic stimulation due to crying in an anxious patient increases oxygen consumption and myocardial work and is poorly tolerated in a child with limited cardiac reserve. Sedative premedication allays anxiety, minimizes O2 consumption and dose of induction agent thereby minimizing reductions in SVR.
11	Induction of GA with titrated doses of anaesthetics, safe use of inhalational agents, in combination with opioids or regional blocks is the best approach.
12	Invasive monitoring to be considered liberally perioperatively in patients with limited hemodynamic reserve and for High-risk surgery.

Anaesthesiologists managing patients with CHD for non-cardiac surgeries should have a proficient knowledge not only of paediatric anaesthesia but also of the pathophysiology of the cardiac lesions and the significance of changes in systemic vascular resistance/pulmonary vascular resistance (SVR/PVR) (Table 2). Risks associated with NCS should be categorized depending on physiological status, disease complexity, type of surgery and age [9]. A thorough preoperative evaluation, focusing on history, physical examination and investigations, details of previous cardiac or NCS including need for prolonged intubation, medication history which may include aspirin, warfarin, antidepressants, diuretics, ACE inhibitors, and anti-arrhythmics, focussing on associated congenital anomalies is of great importance. Recent/current URI, with increased airway reactivity, is poorly tolerated by children with reduced pulmonary compliance. Venous access may be problematic, in children who have had peripheral or central lines in the past. Dehydration should be avoided. Most cardiac medications should be continued preoperatively; aspirin to prevent shunt thrombosis and endocarditis prophylaxis, as indicated. Peri-operative management of hypoxemia, cyanosis, polycythemia, shunt, pulmonary hypertension, and ventricular dysfunction are main considerations. Sympathetic stimulation due to crying due to anxiety and distress can increase oxygen consumption and myocardial work and is poorly tolerated in a child with limited cardiac reserve. Sedative premedication allays anxiety, minimizes O_2_ consumption and dose of induction agent thereby minimizing reductions in SVR. Invasive monitoring is to be considered liberally in patients with limited hemodynamic reserve and for ‘high risk’ surgery [9]. Induction of GA with titrated doses of anaesthetics, safe use of inhalational agents, in combination with opioids or regional blocks is the best approach [8].

In Case 1, history of frequent episodes of URI, poor weight gain, and dyspnea alerted us to the presence and 2D Echo confirmed the cardiac lesion (PDA) in the preoperative period. The patient was categorized into ‘low risk’ category and was operated uneventfully.

Case 2 was a follow-up c/o TAPVC with a Glenn’s Shunt in situ. TAPVC is a cyanotic CHD in which all pulmonary blood flow (PBF) returns to the systemic venous circulation rather than the left atrium. Glenn’s shunt is a type of palliative repair wherein the superior vena cava is anastomosed end-to-side to the (typically right) PA. Thus, systemic venous return from the superior vena cava enters the pulmonary arteries directly. The post-Glenn circulation is characterized by a passive source of PBF, a volume-unloaded ventricle, and persistent cyanosis due to the obligate flow of inferior venacaval return to the RV. Peri-operative anaesthesia concerns included cyanosis, nil cardiorespiratory reserve, ongoing hypoxemia, polycythemia, hyper-viscosity of blood and altered coagulation profile. Goals of anaesthesia remained avoidance of rise in PBF, fall in SVR, dehydration, fever, and rise in hyper-viscosity which can cause cerebral venous and sinus thrombosis. For these reasons dehydration was avoided by allowing the patient clear fluids orally till two hours preoperatively and anti-platelets were continued preoperatively. To allay anxiety and to decrease the dose of induction agent, sedative premedication was administered; to avoid a fall in SVR and for better control of PBF, GA was induced with incremental doses of anaesthetics and a ventilator strategy conducive to the same was instituted. Further, arterial BP was monitored invasively. An ultrasound-guided PEC-1 block was given, at the end of surgery, in addition to opioids and NSAIDS administered routinely, as the sympathetic response to pain, may decompensate the child in the postoperative period. In addition to standard noninvasive monitoring, pain management is very essential in these patients.

Case 3 had an undiagnosed severe valvular AS with bicuspid valve and concentric LVH with bleeding per rectum. AS is the most common cause of left ventricular outflow obstruction in children. A month after the corrective cardiac procedure, patient was taken for emergency NCS as ‘high risk’ (Table 1). The goals of anaesthetic management included avoidance of myocardial ischaemia (avoidance of hypotension and arrythmias), maintenance of SVR, and judicious use of invasive monitoring. Preoperative preparation included blood and fluid resuscitation, IE prophylaxis, minimal sedative premedication, and minimal fasting in addition to psychological support and an informed high-risk consent. Invasive monitoring was used intra-operatively and to avoid a fall in SVR and for better control of PBF, GA was induced with incremental doses of anaesthetics and a ventilator strategy conducive to the same was instituted.

Case 4 was a postoperative c/o TEF, was planned for TOF repair but was meanwhile posted for esophageal dilatation. The association of TEF with prematurity, low birth weight (<1500 g) and CHD has been associated with increased perioperative mortality [11]. It is imperative to repair the TEF at the earliest, even prior to palliation of CHD, as a delay may cause gastric distension resulting in hypoventilation, hemodynamic compromise, and aspiration [11]. The perioperative concerns include ongoing hypoxemia, maintaining a balance between PBF and systemic blood flow [7], preventing cyanotic spells, polycythemia, and coagulopathy in addition to concerns of neonatal age. Peripheral vasodilatation or pulmonary hypertension increase the right to left shunt, and should be prevented by avoiding hypoxia, hypercapnia, acidosis, hypothermia, and by maintaining strict normovolemia [7]. Pulse oximetry overestimates SpO_2_ and EtCO_2_ underestimates PaCO_2_ in cyanotic heart disease patients, this discrepancy increases further with hypoxemia [12], rendering ABG monitoring essential. Postoperative concerns include institution of adequate pain relief, care, and monitoring [3]. Preoperative preparation included starting the patient on oral B-blockers, psychological preparation, and informed high-risk consent. In patients with a right to left shunt, intravenous induction is quicker and is preferred over inhalational induction, ketamine being the preferred agent [7]. The role of precise planning and a coordinated team approach between the intensivist, anaesthesiologist, paediatric surgeon, and physician cannot be over-emphasized in these cases.

Case 5 was the same child as Case 4, posted for a redo-esophageal dilatation post-BT shunt. In addition to the peri-operative anaesthesia concerns which were present earlier, the new concerns were parental psychological distress, an even a greater risk to the child due to poor cardiac condition, cyanosis, hypoxemia, poor weight gain, thrombosed veins, and to fourth exposure of GA. Patient was started on antibiotics preoperatively. Avoidance of cyanotic spells, further hypoxia, hypercapnia, acidosis, and hypothermia were ensured. Normovolemia, psychological preparation and informed high-risk consent were also ensured. After preoxygenation with 100% O2, intravenous induction was carried out with ketamine. Videolaryngoscopy, as the primary plan to aid tracheal intubation along with ABG monitoring were used.

Case 6 was a 15 kg, six-year-old girl with a Glenn’s shunt (in situ), planned for tooth extraction. Major peri-operative concerns included risk of IE, cyanosis, nil cardiorespiratory reserve, ongoing hypoxemia, polycythemia with risk of hyperviscosity and thrombosis. Goals of anaesthesia also included avoidance of rise in PBF and fall in SVR. IE is a life-threatening complication of bacteremia [13,14]. It is now known that routine activities such as toothbrushing and chewing contribute more to the incidence of bacteraemia as compared to dental procedures [13], thereby shifting the focus from antibiotic prophylaxis to greater emphasis on the prevention of oral diseases [14,15]. The 2007 American Heart Association (AHA) guidelines recommended IE prophylaxis mainly for four clinical settings: patients with prosthetic valves; patients who had IE previously; patients with a subset of CHD; and recipients of cardiac transplantation who develop cardiac valvulopathy [13].

Pre-operatively, dehydration was avoided, anti-platelets continued and IE prophylaxis was started. Oral sedative premedication was administered, as is routine. In the OR, the Patient was sedated with IV ketamine and IV midazolam, to avoid a fall in SVR, PVR, and NIBP. Pain management is very essential in these patients, as the sympathetic response to pain may decompensate the child in the postoperative period. IV paracetamol and local anaesthetic infiltration were given for the same.

Limitations

Our study has the inherent limitations of a retrospective observational study. It also represents a small sample size. However, since it is difficult to conduct large scale studies on peri-operative management of such cases, individual experience and expertise will help generate evidence for the management of the same. In addition, it is single centric and experience from multiple centers over different geographical locations would have added more pertinent information on the different types of CHD, various anaesthesia management protocols in these cases according to the institutional or national practices and also, difference according to the local resources. Future research should focus on multicentric and multinational prospective trials on comparison of various anaesthesia techniques in improving outcomes in different subtypes of CHD.

## Conclusions

Children with CHD undergoing NCS have an increased risk of perioperative morbidity. The present study subjects belonged to various risk categories with repaired/unrepaired/palliated CHD and all tolerated anaesthesia well. Based on our experience in this diverse range of CHD cases, ketamine was found to be safe for anaesthesia induction and sevoflurane for maintenance of anaesthesia. Systematic preoperative evaluation, cardiac risk categorization, and optimization, multidisciplinary team approach, judicious utilization of invasive modalities as per the risk category of the patient and multimodal analgesia are the mainstays of management in these patients.
